# The Effect of a 100-km Ultra-Marathon under Freezing Conditions on Selected Immunological and Hematological Parameters

**DOI:** 10.3389/fphys.2017.00638

**Published:** 2017-09-12

**Authors:** Alena Žákovská, Beat Knechtle, Daniela Chlíbková, Marie Miličková, Thomas Rosemann, Pantelis T. Nikolaidis

**Affiliations:** ^1^Department of Animal Physiology and Immunology, Research Centre for Toxic Compounds in the Environment, Institute of Experimental Biology, Faculty of Science, Masaryk University Brno, Czechia; ^2^Institute of Primary Care, University of Zurich Zurich, Switzerland; ^3^Centre of Sports Activities, Brno University of Technology Brno, Czechia; ^4^Exercise Physiology Laboratory Nikaia, Greece

**Keywords:** ultra-runners, 100-km race, hematocrit, leukocrit, immunity

## Abstract

Although moderate exercise is beneficial for the human body and its immune system, exhaustive ultra-endurance performance in cold conditions might be harmful. The aim of this study was to examine the effect of a 100-km ultra-marathon under cold conditions (temperatures from −1°C to +1°C) on selected immunological, biochemical and hematological parameters. Participants were 15 runners (12 men and three women, age 40.3 ± 9.7 years, body mass 67.3 ± 9.0 kg and body height 1.74 ± 0.10 m, mean ± standard deviation). Leukocytes increased (*p* < 0.01) and, particularly, the number of leucocytes doubled in seven out of 15 athletes. Immature neutrophils, mature neutrophils and monocytes increased (*p* < 0.02), whereas lymphocytes and eosinophils did not change. IgG increased (*p* < 0.02), but IgA and IgM remained unchanged. Platelets increased (*p* < 0.01), whereas red blood cells, hematocrit and hemoglobin did not change. lactate dehydrogenase (LDH) and creatine kinase (CK) increased (*p* < 0.01), but alanine aminotransferase (ALT) did not change. There was an association between the markers of the acute inflammation of the organism (i.e., neutrophils, immature neutrophils, platelets, and monocytes) and the markers of muscle damage (i.e., CK, platelets, and LDH). There were no relationships among all the markers in relation to upper respiratory tract infections and liver damage. The highest change was noted in the increase of the number of immature neutrophils (1,019.2%) and CK levels (1,077.6%). In summary, this is the first study investigating immunological, hematological and biochemical parameters and showing that running a 100-km ultra-marathon under cold conditions leads to changes in several immunological, biochemical and hematological parameters indicating a severe stress on the body associated with increasing susceptibility to the development of infections.

## Introduction

Physical activity is recommended for the prevention of various diseases, controlling body mass or delaying the onset of chronic disorders (Pate et al., 1995). On the other hand, metabolic changes associated with prolonged exercise can lead to alterations of blood markers, such as red blood cells decreases, increases in white blood cells, leukocytes and selected immunoglobulins and enzymes (e.g., lactate dehydrogenase, LDH) (Hoffman-Goetz and Pedersen, 1994).

Due to excessive regular training and competitions, modifications in physiological ranges of blood markers of athletes may deviate from their standard physiological state. The total number of white blood cells has been shown to dramatically increase after an ultra-marathon race (Kratz et al., 2002; Smith et al., 2004; Wu et al., 2004). Leukocytosis in runners might be due to an inflammatory response caused by tissue injury. In previous studies (Kratz et al., 2002; Wu et al., 2004; Ramos-Campo et al., 2016), a decrease in the absolute lymphocyte count in the blood of runners was observed immediately after the race, whereas the number of neutrophils and monocytes increased. In addition, red blood cells, hemoglobin and hematocrit have been suggested as screening markers for anemia (Wu et al., 2004). Another blood marker related to exercise and immunity is circulating immunoglobulins. The secretion of immunoglobulin A (IgA) plays a key role in the mucosal immune system providing the “first line of defence” against pathogens (McKune et al., 2005) and has been identified as a marker of mucosal immunity (Gillum et al., 2013).

It is well established that prolonged endurance exercise is associated with muscle cell damage and local inflammation (Armstrong and VanHeest, 2002). According to Nieman and Nehlsen-Cannarella (1991), immunoglobulin M (IgM) may facilitate macrophages in the disposal of muscle cell breakdown products. This could occur by IgM binding to breakdown products present in the blood, followed by their clearance from the circulation. These antibodies may also leave the circulation to carry out the same function in tissues (Nieman and Nehlsen-Cannarella, 1991). Immunoglobulin G (IgG), the most abundant type of antibody, is found in all body fluids and participates in all immune responses (Hořejší et al., 2013). A high level of this antibody indicates an increase in the intensity of immune system activities and suggests an inflammatory reaction (Peters et al., 2010). Other blood markers of immune function might include alanine aminotransferase (ALT), creatine kinase (CK), and lactate dehydrogenase (LDH). The level of LDH has been observed to correlate with both biochemical adaptation to physical load and the state of muscles (Easthope et al., 2010; Janakiraman et al., 2010). The increase in CK is related to both intensity and duration of exercise (Noakes, 1987; Fallon et al., 1999). The above mentioned blood markers have been examined extensively in athletes after a long-term stress race or training under normal temperature conditions (Wu et al., 2004). Variations of crucial markers in response to prolonged exercise under extreme environmental conditions, Cappaert et al. (2008) and Mohammadizadeh et al. (2013), such as very low or very high temperatures, could reflect the development of hyperthermia or hypothermia (Helou et al., 2012; Kälin et al., 2012). According to Sue-Chu (2012), athletes participating in winter sports have a high prevalence of respiratory symptoms.

To date, no study investigated the response of immunological, biochemical and hematological parameters of ultra-marathoners competing under freezing conditions. Such information would be of great theoretical and practical value for exercise physiologists interested in exercise under extreme conditions and coaches working with athletes performing under such conditions, respectively. Therefore, the aim of the present study was to investigate changes in these parameters during 100-km ultra-marathon at ~0°C and we hypothesized that the expected excessive physical load could be reflected in the changes in immunological, biochemical and hematological parameters.

## Methods

### Ethical approval

Ethical approval for the study was obtained from the ethics committee of the Faculty of Science, Masaryk University, Czech Republic.

### Description of the race

The study was conducted during the Czech championship of 100-km ultra-marathon running that was held in Plzeň city (Bohemia) on March 9, 2013. The entire race was on an asphalt surface. The distance of one lap was 1.5 km. The race started at 09:00 a.m. on a flat course at an altitude of 300 m. The temperature varied from −1°C to about +1°C during the race; the wind was ~29 km·h^−1^ in the beginning of the race, but decreased to zero 4 h later. During the race, it snowed and rained continuously. A special tent with drinks, mineral nutrition, fruit, snacks, sugar, salt etc. was available for runners. When the runners wanted to rest, they changed their velocity and instead of running they stopped in the tent for a snack.

### Subjects

The participants were 15–~50% of those who competed in the race: ultra-marathoners (12 men, three women) who ran at least 60 km in the abovementioned 100-km ultra-marathon. They were 40.3 ± 9.7 years old, and had body mass of 67.3 ± 9.0, body height of 1.74 ± 0.10 m and body mass index (BMI) of 22.1 ± 1.2 kg·m^−2^. Their training volume was 91.4 ± 27.0 km·week^−1^ or 10.0 ± 3.7 h·week^−1^. In addition, they competed in such extreme running races at least four times per year. Therefore, they were considered well-trained athletes with 3,679.0 ± 1,270.9 km total training volume during the last year and 40.3 ± 9.7 km as the longest training run before the race.

### Laboratory analysis

The procedures of pre- and post-race blood sampling were identical and were performed at the starting point. The sitting position prior to blood collection was the same in both measurements, since postural changes can influence blood volume and concentration of hematocrit. Blood was collected from an antecubital vein for hematological and chemical analysis using an S-Monovette tube (plasma gel, 7.5 ml) (Sarstedt, Praha, Czech Republic) and an S-Monovette (EDTA, 2.7 ml) (Sarstedt, Praha, Czech Republic) before the start and within 30 min after the race. The collected samples were stored under cold conditions and sent to the laboratory, where they were analyzed within 6 h. Blood samples were obtained to determine red blood cell counts, platelets, hematocrit and hemoglobin. Leukocrit (i.e., the increase in number of leukocytes) and blood differential test were determined using Sysmex XE 2100 (Sysmex Corporation, Japan). The estimation of the enzyme values was performed using reagent sets for determination of LDH/ALT catalytic concentration (ERBA LACHEMA, Brno, Czech Republic). Levels of IgA, IgM, and IgG were determined using assay kits (SENTINEL DIAGNOSTICS, Milano, Italy) for the determination of Ig concentrations. LDH and ALT enzymes and immunoglobulins levels were measured turbidimetrically using spectrophotometer (Rainbow-SLT Instruments, Praha, Czech Republic). CK was determined using Modular SWA (Roche, Basel, Switzerland) by the spectrophotometric method.

### Statistical analysis

All statistical analyses were performed by the statistical package IBM SPSS v.20.0 (SPSS, Chicago, USA). The results were presented as mean and standard deviation (SD). All the data had a normal distribution. Differences between pre- and post-race values were evaluated by paired sample Student's *t*-test. The pre- and post-race values were compared and *p* < 0.05 was accepted as significant.

## Results

Eleven participants finished the 100-km race, whereas four participants did not finish, but completed at least 60 km. The race duration and running pace for each participant are summarized in Table 1. Their body mass did not change during race. The fluid intake during the race was 0.5 ± 0.1 l·h^−1^. The running pace was 6:18 ± 1:42 min·km^−1^. After the race, 11 runners reported weakness, nausea, fever, swelling and muscle spasms.

**Table 1:** Table 1 Race performance of the subjects.

**Athlete**	**Running pace (min/km)**	**Overall time (h:min:s)**	**Completed distance (km)**
1	4:26	7:23:15	100
2	6:01	6:07:40	61
3	7:03	8:23:42	71,5
4	5:26	9:04:01	100
5	7:05	11:40:47	100
6	5:42	9:29:18	100
7	6:59	11:38:24	100
8	6:17	10:28:21	100
9	7:50	12:03:55	92.5
10	9:18	10:22:58	67
11	4:37	7:41:54	100
12	6:23	10:38:47	100
13	5:50	9:43:21	100
14	7:08	11:51:47	100
15	5:49	9:41:48	100

### Immunoglobulins

**Figure 1:**
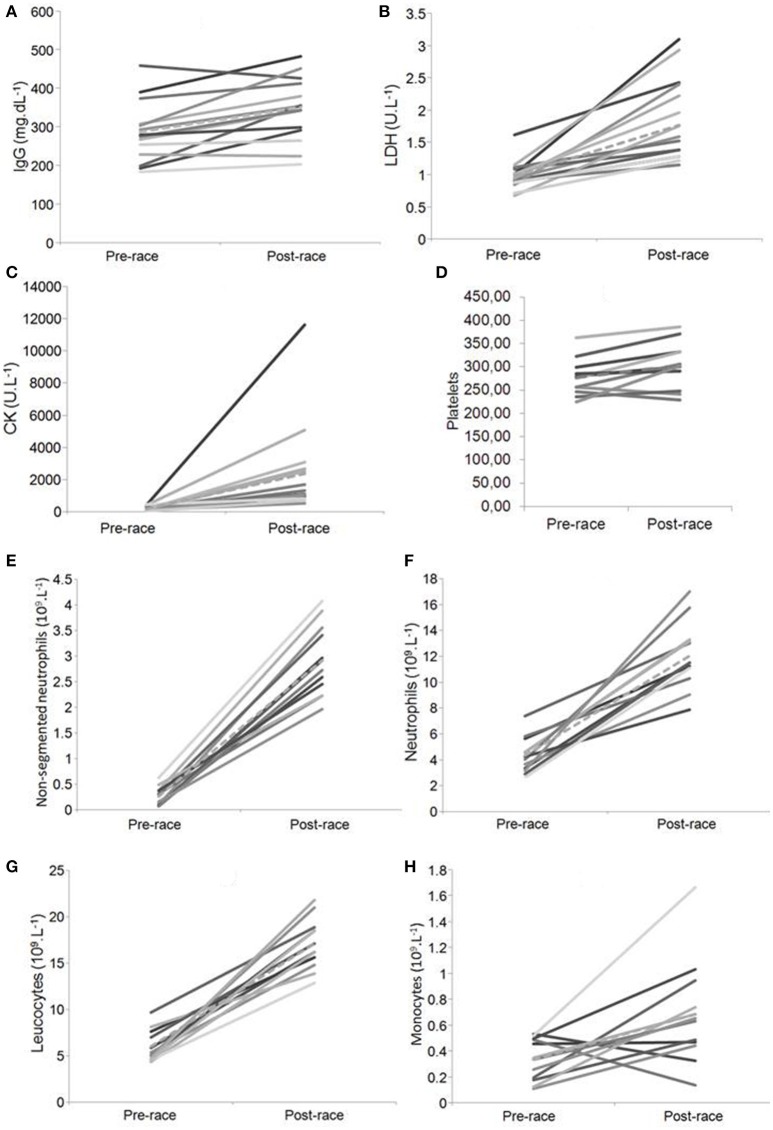
Statistically significant changes in pre- and post-race levels of IgG, LDH, CK, platelets, number of non-segmented (immature) neutrophil granulocytes, neutrophil granulocytes, leukocytes ^*^Note. Dashed line–Mean value; **(A)** IgG, **(B)** LDH, **(C)** CK, **(D)** Platelets, **(E)** Non-segmented (immature) neutrophils, **(F)** Neutrophils, **(G)** Leukocytes, **(H)** Monocytes.

Pre- and post-race serum immunoglobulins, IgA and IgM (Table 2), were within clinical reference ranges. IgG increased post-race by 22.6% (*p* < 0.02; Figure 1A) and it exceeded the normal reference range in some cases.

**Table 2:** Comparison of immunological, hematological, and biochemical parameters before and after the race (Mean ± *SD* and % change) without statistically significant changes.

**Parameter**	**Before the race**	**After the race**	**% change**
Red blood cells (10^12^·l^−1^)	4.84 (0.31)^a^	4.81 (0.32)^a^	−0.62
Hematocrit	0.43 (0.02)^a^	0.42 (0.02)^a^	−0.94
Hemoglobin (g·dl^−1^)	135.27 (15.72)^a^	133.43 (16.57)^a^	−1.36
Alanine aminotransferase (U·l^−1^)	8.91 (4,17)^c^	8.39 (5.17)^c^	−5.84
Lymphocytes (10^9^·l^−1^)	1.40 (0.28)^a^	1.41 (0.46)^a^	0.71
Eosinophils (10^9^·l^−1^)	0.12 (0.17)^a^	0.04 (0.08)^a^	−66.67
Basophils (10^9^·l^−1^)	0	0	0.00
Immunoglobulin A (mg·dl^−1^)	21.09 (90.52)^c^	24.0 (13.56)^c^	13.80
Immunoglobulin M (mg·dl^−1^1)	24.72 (136.15)^c^	24.78 (137.00)^c^	0.24

Number of participants (after the race, bad statue of some of the respondents resulted in problems with blood intake for hematological analysis):

a (12);

b (14);

c (15); % change—percentage change of variables (the difference between pre- and post-quantity)

### Blood cells

The leukocytes increased post-race by 185.3% (*p* < 0.01; Figure 1G) and exceeded the reference range in all participants. The segmented (i.e., mature neutrophils) and non-segmented neutrophils (i.e., immature neutrophils) granulocytes increased post-race by 178.1 and 1,019.2%, respectively (*p* < 0.01; Figures 1E,F,). The monocytes increased by 153.3% (*p* < 0.02; Figure 1H), whereas the lymphocytes and eosinophils showed no changes (*p* > 0.05; Table 2). The erythrocytes, hemoglobin, and hematocrit were within the normal range pre-race. The platelets increased post-race by 9.4% (*p* < 0.01; Figure 1D), whereas the number of erythrocytes, hemoglobin and hematocrit did not change (Table 2).

### Enzymes alanine aminotransferase, lactate dehydrogenase, and creatine kinase

Lactate dehydrogenase increased post-race by 79.6% (*p* < 0.01; Figure 1B), whereas ALT did not change (*p* > 0.05; Table 2). CK levels increased by 1,077.6% (*p* < 0.01; Figure 1C).

### Dependence on variables

Parameters indicate the average percentage change before (PRE) and post-race (POST) and the statistical significance of this change. Statistical data show a relationship among all the markers in relation to the inflammation of the organism (i.e., neutrophils, immature neutrophils, platelets and monocytes) (see individual variability in Figures 1D–F,H); to muscle damage (i.e., CK, platelets and LDH) (see individual variability in Figures 1B–D); there were no relationships between the markers and both upper respiratory tract infections (URTI) [i.e., leukocrit, IgA and IgM (Table 2)] and liver damage [i.e., LDH, ALT (Table 2)].

## Discussion

The present study examined changes in immunological and physiological parameters in runners competing to a 100-km ultra-marathon under cold and windy conditions. In the Czech Republic, the racing season for athletes and runners is usually around the spring and summer months. Therefore, Czech runners are accustomed to run and participate in the races under warm conditions. The described race was held at the end of winter time, in cold weather. This is the time period in which runners prepare for the spring and summer season, and undergo hard training. They are not accustomed to participating in races under such cold conditions and because of that, they are not sufficiently relaxed.

The main findings were that (i) after the race, leukocytes increased and in seven of 15 athletes the number of leucocytes doubled; (ii) immature neutrophils, mature neutrophils and monocytes increased, whereas the number of lymphocytes and eosinophils did not change; (iii) IgG increased, but IgA and IgM remained unchanged; (iv) platelets increased, whereas the number of red blood cells, hematocrit and hemoglobin did not change; and (v) LDH and CK values increased, but ALT concentration did not change.

### Leukocrit

A major discovery was that during the race, leukocytes increased above the reference range. This was in agreement with previous studies (Smith et al., 2004; Wu et al., 2004; Lombardi et al., 2013; Shin and Lee, 2013). The increase of leukocytes might be due to a mechanism described by Nielsen et al. (2004), according to whom strenuous exercise leads to an increase of catecholamine that is responsible for the recruitment of marginated leukocytes (Nielsen et al., 2004) resulting in the release of leukocytes from the bone marrow.

Another explanation might be that of Smith et al. (2004) and Gleeson et al. (2013), who assumed that leucocytosis observed in runners may occur due to an inflammatory response caused by a tissue injury. This tissue injury correlates with both intensity and duration of exercise (Smith et al., 2004), which is consistent with our results showing a 185% increase in the number of leukocytes, wherein the total number of neutrophils, non-segmented (immature) neutrophils and monocytes increased. A similar result in the increase of neutrophils, non-segmented (band) neutrophils and monocyte was also observed by the studies of Kratz et al. (2002), Rama et al. (2016), and Wu et al. (2004), where the increase in white blood cells count was mainly due to neutrophilia and monocytosis, with a relative decrease in circulating lymphocytes, consistent with an inflammatory reaction to tissue injury. Platelet, monocyte and neutrophil counts increased during inflammation (Herter et al., 2014). Our outcome manifested similar results. For that, we could state, there exists a relationship between the statistically significant increases in platelets, monocytes and neutrophils and the process of an acute inflammation.

The increase in neutrophils and non-segmented neutrophils after severe exercise may be promoted by mechanisms reported by Rippe (2011). First, the elevation of the neutrophils count could be enhanced by neutrophils' demargination from endothelial walls. This is caused by the elevation of epinephrine level in plasma. Second, the increase of non-segmented neutrophils could be explained by the release and margination of these immature neutrophils from bone marrow to blood in the action of glucocorticoids. Krejsek and Kopecký (2004) and Rama et al. (2016) describe the way intense and long exercise results in an alteration and elevation of leukocytes and changes in differential blood count. They asserted the occurence of an increase of both neutrophils and their immature forms after intensive exercise, which is consistent with our results. It was found that an elevated amount of neutrophils along with their immature forms, eosinophils and monocytes are mobilized to enter the peripheral blood during ongoing severe long-distance exercise, and lymphocytes decrease after prolonged exercise (Krejsek and Kopecký, 2004; Rama et al., 2016). Our study confirms that participating in a 100-km ultra-marathon requires long-term, regular, and high-volume training; however, even such good preparation cannot anticipate changes in the number and type of leukocytes (immature neutrophils, neutrophils, and monocytes).

### Immunoglobulins

We examined the total amount of IgM, IgA, IgG antibodies. Circulating immunoglobulins are generally associated with humoral adaptive immunity (Hořejší et al., 2013). We observed an increase in the post-race level of IgG, which was in agreement with previous research on similar or shorter race distance (Mackinnon, 1999; Hoffman, 2002), which showed that IgG and IgM levels increased after a short maximal exercise. A higher production of IgG level after a similar ultra-marathon race was reported by McKune et al. (2005). An increased IgG was also reported by Karacabey et al. (2005) after aerobic and anaerobic exercise. The appearance of an inflammatory response was indicated by several markers, such as increased leukocytes and platelets (Karacabey et al., 2005).

The starting process of an inflammatory response might be due to the increased IgG, though lymphocytes did not change. Other underlying mechanisms that might also contribute to the increase of IgG levels include the increase of catecholamines, neuropeptides and the stress hormone cortisol highlighting the major role of central nervous system. The increase of IgG is a secondary consequence of the release of the abovementioned hormone substances (Hejazi and Hosseini, 2012). Relevant studies examining changes in immunoglobulin responses did not show similar results; some studies showed changes whereas others did not. For instance, our results were in agreement with the study of Gunzer et al. (2012), claiming that serum Ig concentrations are unaffected or only slightly increased, except for a decline in salivary IgA concentration and secretion rate. According to Karacabey et al. (2005), the increasing level of immunoglobulins after a race might have a protective effect. The increase of IgG might be promoted by switching from IgM to IgG. This phenomenon is regulated by certain cytokines, which are produced by TH2 lymphocytes (Il-4, Il-6) and cortisol (McKune et al., 2005). On the other hand, the number of B-cells does not change in response to exercise (Rippe, 2011).

Another major discovery was that IgM, which is the largest antibody in the human circulatory system, did not increase in the participants. IgM is the first antibody to act in response to initial exposure to antigen (Trochimiak and Hübner-Woźniak, 2012). The changes in IgM levels after an ultra-distance exercise might be due to the activation of the classical complement pathway, which might be activated during tissue injury because of the intense and prolonged exercise. Since IgM moves into the tissues, where it cleaves destroyed cells with the cooperation of macrophages, it is not possible to detect IgM in its full amount in the blood serum (Nieman and Nehlsen-Cannarella, 1991).

With regard to IgA, which is an immunoglobulin that neutralizes pathogens that have crossed the mucosal barrier, no change was observed. Many previous studies examined post-race levels of IgA (Walsh et al., 1999; Libicz et al., 2006; Trochimiak and Hübner-Woźniak, 2012). A decreased level of IgA could be a possible reason for infections of the upper respiratory tract in ultra-distance athletes after excessive exercise, according to Trochimiak and Hübner-Woźniak (2012). Nieman and Pedersen (1999) reported that the increased risk of infection of the upper respiratory tract is possible after intense bouts of long duration exercise of more than 2 h, such as a marathon or an ultra-marathon. In our study, the statistical analysis of markers, such as leukocrit, IgM and IgA to the possible occurrence of URTI revealed no relationship. Rippe (2011) observed increases in the amount of circulating neutrophils, monocytes, natural killer cells, and a higher concentration of several plasma hormones, such as cortisol and growth hormone after an ultra-marathon race. An excessive and prolonged load accompanied by poor nutrition, lack of sleep and psychological stress are crucial factors contributing to the decrease in IgA levels and significant changes in other pro-inflammatory markers (Walsh et al., 2011). The duration of the time demanded for recovery of an organism after such an exhaustive race depends on the individuals themselves. The approximate time for an immune-regeneration lasts from 3 to 72 h (Gunzer et al., 2012), but in some athletes, this time may be several weeks (Gleeson, 2007; Gleeson et al., 2011). Vitamin D deficiency is another factor which can play a role in developing URTI and appears to have an important influence on mucosal immunity of endurance athletes. Our race took place in the beginning of March, so the decreased levels of IgA in some of our measured athletes might reflect the lack of vitamin D after winter (He et al., 2013).

### Hematocrit

Red cells, hemoglobin and hematocrit, three potential indicators of anemia, were normal before and after the race in the present study. Ultra-marathons are also associated with a wide range of significant changes in hematological parameters (Kratz et al., 2002; Nielsen et al., 2004; Lombardi et al., 2013). The ubiquitous presence of platelets and their ability to modulate early inflammation suggests a vital role in the acute inflammatory phase (Herter et al., 2014). The platelet count in our findings increased during the race. In the study of Wu et al. (2004), an elevated number of platelets was observed only after the ninth day of the race. Similar results were revealed by the study of Rama et al. (2016) where the increased amount of platelets was shown after the fifth stage of an ultra-marathon. In our results, an increased number of platelets appeared immediately after the race, and their increase correlates with an increase in the amount of mature and immature neutrophils and monocytes as the first inflammatory effectors. According to Wu et al. (2004), red cell count, hemoglobin and hematocrit levels did not decrease immediately after a race, but decreased from 2 to 9 days after a race in accordance with the damage of red blood cells due to endurance exercise. The so-called “sports anaemia” is not only caused by intravascular haemolysis due to mechanical trauma, but also due to oxidative injuries of red blood cells (Selby and Eichner, 1986). Unfortunately, we had no opportunity to test the parameters of runners at longer intervals after the race.

### Other biochemical parameters

Creatine kinase (CK) increased after the race by 1,077.6%. One of the main causes of this rise could be the extreme duration of this race of (8–12 h). Noakes (1987) reported that each runner possibly shows a maximum increase in serum enzyme activity, which may be achieved by running for 4–6 h. According to Fallon et al. (1999) and Noakes (1987), the increase of CK is related to both intensity and duration of exercise. Normally, CK plays a significant role inside the cells, and its concentration in whole blood is low (Fedotovskaya et al., 2012). CK is a key enzyme in energy creation for skeletal muscle performance. An excessive increase of CK level in blood is a predominant trait of a marked physical stress (Marklund et al., 2013). CK along with LDH are the main traits of muscle injury, and this result is also consistent with our study, supported by a statistically significant change in platelets. A concentration of CK from 167 to 3,334 μkat·l^−1^ signals rhabdomyolysis (Efstratiadis et al., 2007); the CK of participants in the present study were within this range.

Lactate dehydrogenase (LDH), an enzyme located inside erythrocytes and myocytes, increased post-race significantly, which agreed with previous studies on other ultra-marathon events (Kanter et al., 1986; Nagel et al., 1990; Fallon et al., 1999). According to these studies, post-race enzyme levels may rise far beyond reference values as a result of strenuous exercise. Thus, the concentration of LDH in the whole blood is minimal. Intense exercise may result in release of LDH from muscle cells and erythrocytes to raise the concentration in blood. This may support the theory about intravascular haemolysis and skeletal muscle damage after a 100-km ultra-marathon (Easthope et al., 2010; Janakiraman et al., 2010). LDH levels may also be increased by haemolysis, involving elevated bilirubin levels. There are several explanations for possible ongoing haemolysis including osmotic stress or membrane lipid peroxidation caused by free radicals released by activated leukocytes (Fallon et al., 1999).

Alanine aminotransferase (ALT) concentration was not elevated after the race, indicating no liver damage, which was in agreement with the study of Deugnier et al. (2002), which highlighted that accurate assessment and interpretation of ALT concentrations in professional and nonprofessional athletes are essential for diagnosis and prevention. The enzyme ALT catalyzes in the liver the formation of pyruvate and glutamate. It is predominantly located in cytoplasma of hepatocytes and myocytes (Bürger-Mendonça et al., 2008). Out-of-range ALT levels provide indirect evidence of possible hepatic inflammation or damage during prolonged exercise. In our study there was no liver damage when compared to statistically significant parameters in ALT and LDH.

From the 17 measured parameters, eight show differences between pre- race and post- race values. A *p*-value lower than 0.01 was observed in six of these parameters (i.e., leukocytes, neutrophils, monocytes, IgG, LDH, and CK). Some of these parameters play a role in both specific and non-specific immunity. The first activated immune cells are monocytes and both types of neutrophils. They have a key role in the first defense line of immunity. This process causes the inflammatory response after tissue injury (Kratz et al., 2002; Rama et al., 2016). On the basis of the resulting and developing inflammation process and enhanced immune response (McKune et al., 2005), an increased level of IgG in athletes was observed, although the number of lymphocytes remained the same. The indicators of muscle tissue damage, including CK and LDH enzymes, also manifested a rise after the race (Efstratiadis et al., 2007). The above mentioned change supports the theory of the emergence and development of an inflammatory response in the body of an athlete, which could lead to the damage of certain parts of the musculoskeletal system and the emergence of a temporary state of immunosuppression.

### Cold weather

In the Czech Republic, the racing season for athletes and runners is usually around the spring and summer months. Therefore, Czech runners are accustomed to run and participate in the races under warm conditions. The described race was held at the end of winter time in cold weather. This is the time period in which runners prepare for the spring and summer season and undergo hard training. They are not accustomed to participating in races under such cold conditions and because of that, they may not be well prepared for the race. So far, an insufficient number of studies have elaborated on this topic in the past. Those available studies suggest that extremely cold or hot temperatures have deleterious consequences for the immune system of participants and for their athletic performance (Cappaert et al., 2008). Despite the fact that concrete consequences arising from prolonged running races held in excessively cold or hot conditions vary, we propose that running performance and race speed is more affected and reduced during extremely hot temperatures than in cold ones (Helou et al., 2012). However, marathon races in cold weather could result in the increased susceptibility to bronchoconstriction, chest tightness, cough, exercise-induced asthma or excessive production of mucous fluid (Mohammadizadeh et al., 2013). In addition, several cold injuries related to frostbite, such as Trench foot, hypothermia or chilblain, may also be easily developed in participants of such extreme races (Cappaert et al., 2008). Sue-Chu (2012) revealed that inflammatory response, which could be reflected in the rise in influx of neutrophils and respiratory pathway epithelial damage, could be the result of persevering exercise accompanied by cold temperatures. He also claims that during these hostile conditions, hyperpnoea could more easily develop in the participants.

Based on previous findings in runners competing at moderate temperatures, we hypothesized that the parameters under consideration would change to a greater extent considering the additional physiological impact of cold. The influence of cold along with the severe physical load could be reflected in the physical state of the runners after the race, which includes weakness, nausea, fever, swelling, and muscle spasms (Cappaert et al., 2008; Helou et al., 2012). Although they were well prepared for the long run, 11 runners reported weakness, nausea, fever, swelling and muscle spasms. According to our results, the emergence of muscle spasms is supported by the association between CK, platelets and LDH with skeletal muscle damage, as the cold environmental conditions could affect a few parameters. When we compared our results with the results of running a marathon in a climate chamber with extremely wide temperature ranges (−45°C to +55°C) (Kälin et al., 2012), we observed similar results. The post-race concentrations of leukocrit, platelet count and blood differential were analogous. No changes in hemoglobin and hematocrit were shown and from biochemical parameters, both LDH and CK levels increased (Kälin et al., 2012). Participants in the present study, as well as in that of Kälin et al. (2012), competed under very unfavorable environmental conditions, which were reflected in the monitored markers.

The results of the present study are limited by the race distance, because the observed changes in physiological markers of exercise might vary in shorter or larger distances. The race under examination is one of the longest ultra-marathons in the Czech Republic, but not worldwide. Thus, caution is needed to generalize the findings to other distances. Furthermore, a limitation of the present study was that it had a pre-experimental design, particularly, a one-group pre-test-post-test design. In a true experimental model, i.e., pre-test-post-test randomized-groups model, participants would compete in both cold and normal temperatures. However, such an optimal design was not feasible. Athletic performance during the race could be influenced by three major factors: the first one regards overall athletic health (the state of the immune system, age, gender, athletic event experience and preparation, etc.). The second one points out the role of the genetic factor. The last one deals with environmental conditions and includes factors, such as air temperature, air pollution, wind speed, etc. Out of all of these environmental factors, the most significant is the air temperature (Helou et al., 2012). On the other hand, a strength of the study is its novelty, since, to the best of our knowledge, it is the first to examine the combined effect of both 100-km running and cold environment. Although previous studies examined physiological responses during ultra-endurance exercise in cold (Cappaert et al., 2008; Helou et al., 2012; Kälin et al., 2012), the present study is the first to examine the exercise-induced changes of immunological, biochemical, and blood markers in such environmental conditions. It suggests that by increasing the number of innate immune cells under these conditions, the body undergoes an acute inflammation process. These findings are of great theoretical interest for exercise physiologists as exercise in extreme cold is a major topic of exercise physiology. Furthermore, they have practical applications for coaches and runners to improve their training and competition, considering the specific physiological demands of the specific exercise and environmental conditions.

## Conclusions

In summary, a wide range of physiological changes in athletes occurred after a 100-km ultra-marathon under cold conditions. We concluded that prolonged endurance running had a negative effect on the immune system expressed as inflammation. Moreover, it could affect physiological functions of the body which could exhibit elevated susceptibility to the development of infection. Long exposure to cold weather along with the length and severity of the race, our respondents' athletic preparation and their healthy state or profile of the track might contribute to the significant changes leading to inflammation of the body and muscle damage. This can be reflected in the increase of platelet count and extreme increase of non-segmented neutrophils which play a role in the acute inflammation phase along with the elevated CK level.

## Author contributions

AZ drafted the manuscript. AZ, MM, and DC performed the experiments. BK, TR and PN helped in drafting the manuscript.

### Conflict of interest statement

The authors declare that the research was conducted in the absence of any commercial or financial relationships that could be construed as a potential conflict of interest.
